# Supplementary figures and images for: Nutritional composition and antioxidant properties of the fruit of Berberis heteropoda Schrenk
Source: PLoS One. 2022 Apr 7;17(4):e0262622. doi: 10.1371/journal.pone.0262622 (PMC8989241; doi:10.1371/journal.pone.0262622)

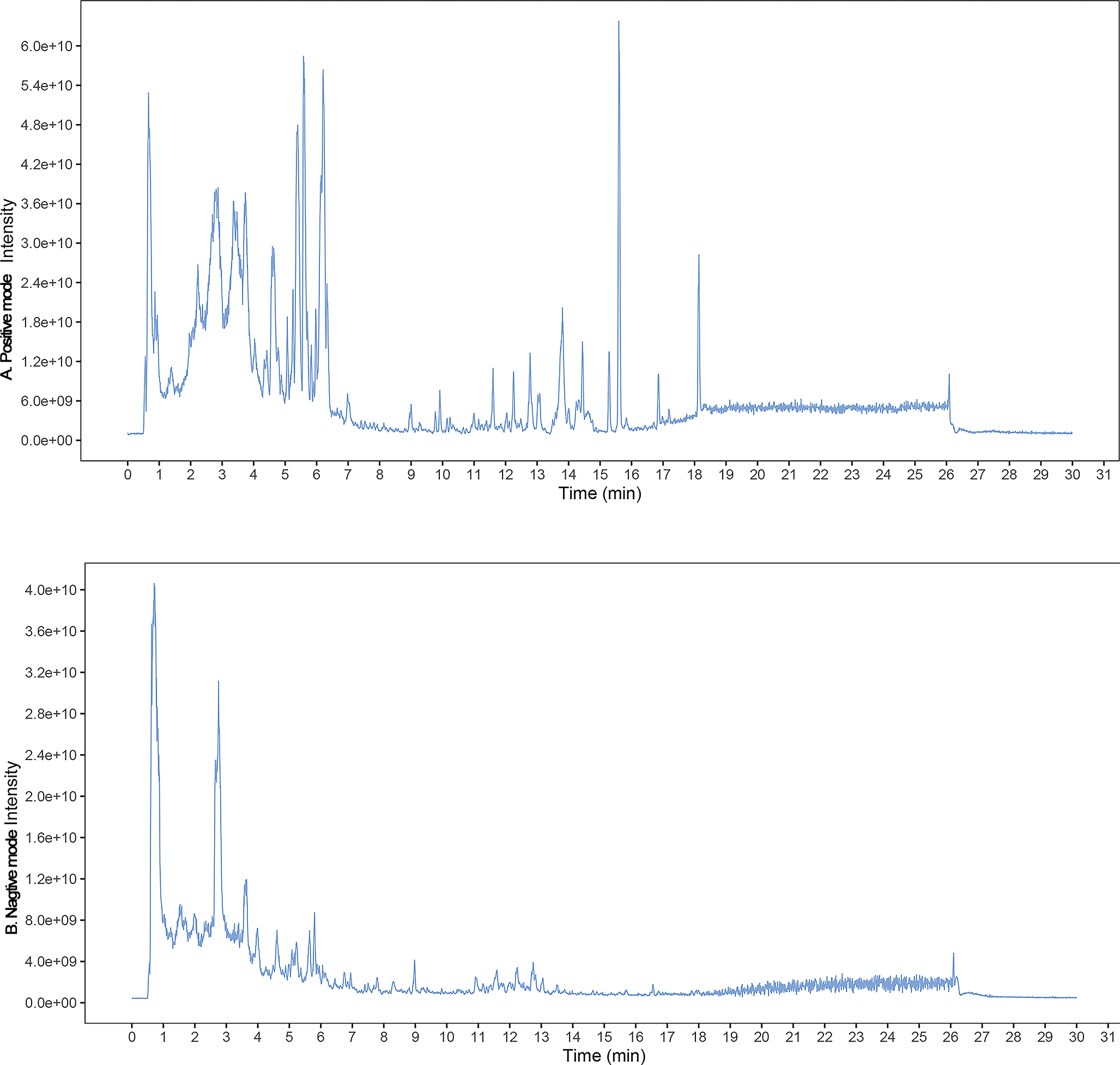

Supplement: S1 Fig — (TIF) [file pone.0262622.s001.tif]
