# Supplementary material for: Nutritional composition and antioxidant properties of the fruit of Berberis heteropoda Schrenk
Source: PLoS One. 2022 Apr 7;17(4):e0262622. doi: 10.1371/journal.pone.0262622 (PMC8989241; doi:10.1371/journal.pone.0262622)

PLOS ONE

Nutritional Composition and Antioxidant Properties of the Fruit of Berberis heteropoda Schrenk

--Manuscript Draft--

**Manuscript Number:**	PONE-D-21-29715
**Article Type:**	Research Article
**Full Title:**	Nutritional Composition and Antioxidant Properties of the Fruit of Berberis heteropoda Schrenk
**Short Title:**	Nutrition and antioxide of Berberis heteropoda Schrenk
**Corresponding Author:**	Jianguang Li<br>Xinjiang Medical University<br>Urumqi, CHINA
**Keywords:**	Berberis heteropoda Schrenk; nutritional composition; antioxidant properties; phenol; flavonoid
**Abstract:**	Objective<br><br>This study aimed to assess the major nutrients and antioxidant properties of Berberis heteropoda Schrenk fruits collected from the Nanshan Mountain area of Urumqi City, Xinjiang Uygur Autonomous Region, China.<br><br>Methods and Materials<br><br>Basic nutrients including amino acids, minerals, and fatty acids were assessed, and the total phenol, flavonoid, and anthocyanin contents of the extracts were determined.<br><br>Results<br><br>The analytical results showed that average water, total fat, total protein, ash, and carbohydrates contents in Berberis heteropoda Schrenk fruits were 75.22, 0.506, 2.55, 1.31, and 17.72 g100 g fresh fruit, respectively. The total phenol, flavonoid, and anthocyanin contents of Berberis heteropoda Schrenk fruits were 68.55 mg gallic acid equivalentsg, 108.42 mg quercetin equivalentsg, and 19.83 mg cyanidin-3-glucoside equivalentg, respectively. The UPLC-Q-TOF-MSE analysis of phenols revealed 32 compounds.<br><br>Conlusions<br><br>The findings suggest Berberis heteropoda Schrenk fruitsmay have potential nutraceutical value and can be considered a potential source of nutritional components and antioxidant phytochemicals in the human diet.
**Order of Authors:**	Jixiang Sun
	Qian Li
	Jianguang Li
	Jing Liu
	Fang Xu
**Additional Information:**	
**Question**	**Response**
**Financial Disclosure**<br><br>Enter a financial disclosure statement that describes the sources of funding for the work included in this submission. Review	This research was funded by the Research Foundation of Xinjiang Uygur Autonomous Region (Natural Science Foundation) Joint Fund Project, grant number 2021D01C177.

Powered by Editorial Manager® and ProduXion Manager® from Aries Systems Corporation

the submission guidelines for detailed requirements. View published research articles from *PLOS ONE* for specific examples.<br><br>This statement is required for submission and **will appear in the published article** if the submission is accepted. Please make sure it is accurate.<br><br>**Unfunded studies**<br>Enter: *The author(s) received no specific funding for this work.*<br><br>**Funded studies**<br>Enter a statement with the following details:<br>• Initials of the authors who received each award<br>• Grant numbers awarded to each author<br>• The full name of each funder<br>• URL of each funder website<br>• Did the sponsors or funders play any role in the study design, data collection and analysis, decision to publish, or preparation of the manuscript?<br>• **NO** - Include this sentence at the end of your statement: *The funders had no role in study design, data collection and analysis, decision to publish, or preparation of the manuscript.*<br>• **YES** - Specify the role(s) played.<br><br>* typeset	
**Competing Interests**<br><br>Use the instructions below to enter a competing interest statement for this submission. On behalf of all authors, disclose any competing interests that could be perceived to bias this work—acknowledging all financial support and any other relevant financial or non-financial competing interests.<br><br>This statement is required for submission and **will appear in the published article** if the submission is accepted. Please make sure it is accurate and that any funding sources listed in your Funding Information later in the submission form are also	The authors have declared that no competing interests exist.

declared in your Financial Disclosure statement.<br><br>View published research articles from *PLOS ONE* for specific examples.<br><br>**NO authors have competing interests**<br><br>Enter: *The authors have declared that no competing interests exist.*<br><br>**Authors with competing interests**<br><br>Enter competing interest details beginning with this statement:<br><br>*I have read the journal's policy and the authors of this manuscript have the following competing interests: [insert competing interests here]*<br><br>* typeset	
**Ethics Statement**<br><br>Enter an ethics statement for this submission. This statement is required if the study involved:<br><br>• Human participants<br>• Human specimens or tissue<br>• Vertebrate animals or cephalopods<br>• Vertebrate embryos or tissues<br>• Field research<br><br>Write "N/A" if the submission does not require an ethics statement.<br><br>General guidance is provided below. Consult the submission guidelines for detailed instructions. **Make sure that all information entered here is included in the Methods section of the manuscript.**	N/A

Powered by Editorial Manager® and ProduXion Manager® from Aries Systems Corporation

**Format for specific study types**<br><br>**Human Subject Research (involving human participants and/or tissue)**<br>• Give the name of the institutional review board or ethics committee that approved the study<br>• Include the approval number and/or a statement indicating approval of this research<br>• Indicate the form of consent obtained (written/oral) or the reason that consent was not obtained (e.g. the data were analyzed anonymously)<br><br>**Animal Research (involving vertebrate animals, embryos or tissues)**<br>• Provide the name of the Institutional Animal Care and Use Committee (IACUC) or other relevant ethics board that reviewed the study protocol, and indicate whether they approved this research or granted a formal waiver of ethical approval<br>• Include an approval number if one was obtained<br>• If the study involved *non-human primates*, add *additional details* about animal welfare and steps taken to ameliorate suffering<br>• If anesthesia, euthanasia, or any kind of animal sacrifice is part of the study, include briefly which substances and/or methods were applied<br><br>**Field Research**<br><br>Include the following details if this study involves the collection of plant, animal, or other materials from a natural setting:<br>• Field permit number<br>• Name of the institution or relevant body that granted permission	
**Data Availability**<br><br>Authors are required to make all data underlying the findings described fully available, without restriction, and from the time of publication. PLOS allows rare exceptions to address legal and ethical concerns. See the PLOS Data Policy and FAQ for detailed information.	Yes - all data are fully available without restriction

A Data Availability Statement describing where the data can be found is required at submission. Your answers to this question constitute the Data Availability Statement and **will be published in the article**, if accepted.<br><br>**Important:** Stating 'data available on request from the author' is not sufficient. If your data are only available upon request, select 'No' for the first question and explain your exceptional situation in the text box.<br><br>Do the authors confirm that all data underlying the findings described in their manuscript are fully available without restriction?	
**Describe where the data may be found in full sentences. If you are copying our sample text, replace any instances of XXX with the appropriate details.**<br><br>• If the data are **held or will be held in a public repository**, include URLs, accession numbers or DOIs. If this information will only be available after acceptance, indicate this by ticking the box below. For example: *All XXX files are available from the XXX database (accession number(s) XXX, XXX.).*<br>• If the data are all contained **within the manuscript and/or Supporting Information files**, enter the following: *All relevant data are within the manuscript and its Supporting Information files.*<br>• If neither of these applies but you are able to provide **details of access elsewhere**, with or without limitations, please do so. For example:<br><br>*Data cannot be shared publicly because of [XXX]. Data are available from the XXX Institutional Data Access / Ethics Committee (contact via XXX) for researchers who meet the criteria for access to confidential data.*<br><br>*The data underlying the results presented in the study are available from (include the name of the third party*	All relevant data are within the manuscript and its Supporting Information files.

and contact information or URL).<br>• This text is appropriate if the data are owned by a third party and authors do not have permission to share the data.<br><br>* typeset	
Additional data availability information:	

Powered by Editorial Manager® and ProduXion Manager® from Aries Systems Corporation

Manuscript Click here to access/download;Manuscript;Manuscript.docx

Nutritional Composition and Antioxidant Properties of the Fruit of *Berberis heteropoda* Schrenk

Short title: Nutrition and antioxide of *Berberis heteropoda Schrenk*

Jixiang Sun[1,2], Qian Li [2], Jianguang Li[1,*], Jing Liu[2], and Fang Xu[1]

[1]College of Pharmacy, Xinjiang Medical University, Urumqi 830011, China

[2]Clinical Nutrition Institute of Xinjiang Uygur Autonomous Region People's Hospital, Urumqi 830001, China;

*Correspondence:

Jianguang Li, Ph.D.

College of Pharmacy, Xinjiang Medical University,

No.393, Xinyi Road, Urumqi 830001, China

Email: xjykdx_ljg@163.com;

Tel: +86-991-4363000

ABSTRACT

Objective: This study aimed to assess the major nutrients and antioxidant properties of *Berberis heteropoda* Schrenk fruits collected from the Nanshan Mountain area of Urumqi City, Xinjiang Uygur Autonomous Region, China.

Methods and Materials: Basic nutrients including amino acids, minerals, and fatty acids were assessed, and the total phenol, flavonoid, and anthocyanin contents of the extracts were determined.

Results: The analytical results showed that average water, total fat, total protein, ash, and carbohydrates contents in *Berberis heteropoda* Schrenk fruits were 75.22, 0.506, 2.55, 1.31, and 17.72 g100 g fresh fruit, respectively. The total phenol, flavonoid, and anthocyanin contents of *Berberis heteropoda* Schrenk fruits were 68.55 mg gallic acid equivalentsg, 108.42 mg quercetin equivalentsg, and 19.83 mg cyanidin-3-glucoside equivalentg, respectively. The UPLC-Q-TOF-MSE analysis of phenols revealed 32 compounds.

Conlusions: The findings suggest *Berberis heteropoda* Schrenk fruitsmay have potential nutraceutical value and can be considered a potential source of nutritional components and antioxidant phytochemicals in the human diet.

Keywords: *Berberis heteropoda* Schrenk; nutritional composition; antioxidant properties; phenol; flavonoid

Introduction

Berberis heteropoda Schrenk is a shrub of the family Berberidaceae and is distributed in the Altai, Tianshan, and Baluke mountains of the Xinjiang Uygur Autonomous Region, China, as

well as in Mongolia and Kazakhstan [1]. The roots, bark, stems, and fruits of *Berberis heteropoda* Schrenk are traditionally used as herbal medicine, and the fruits in particular have historically been consumed as tea [2,3]. Studies have confirmed traditional beliefs, suggesting that this fruit can be used to treat dysentery, enteritis, pharyngitis, stomatitis, eczema, and hypertension [4,5]. The nutritional and antioxidant properties of *Berberis heteropoda* Schrenk fruit are significantly related to the distribution of molecular and secondary metabolites, which should be understood to obtain benefits from its consumption.

In addition to protein, fat, dietary fiber, minerals, and other nutrients, plants also contain numerous phenolic components that play an important role in human health [6]. Polyphenols are secondary metabolites produced by plants ~~during growth and~~ are widely found in vegetables, fruits, forages, ~~and medicinal plants~~ [7]. Phenolic compounds are effective in preventing oxidation at the cellular and physiological levels, and their antioxidant capacity is determined based on the arrangement of hydroxyl and carbonyl groups in their structures, as well as the gain and loss of electrons from hydrogen atoms to reduce free radicals and form stable phenoxy groups [8,9]. Flavonoids are the major components of plant polyphenols, which with high redox plays an important role in antioxidant effects, including reduction, hydrogen donor, singlet oxygen quenching, and metal chelation. Thus, evaluating the content of polyphenols and flavonoids and antioxidant capacity of *Berberis heteropoda* Schrenk fruit and developing its medicinal and nutritional value are important.

~~While~~ recent studies have focused on the anthocyanin composition of *Berberis heteropoda* Schrenk fruit [3], the nutritional composition and phenolic components remain less understood. Therefore, we conducted this study to assess the major nutrients and their

antioxidant properties of *Berberis heteropoda* Schrenk, providing additional information regarding the nutritional roles and health benefits of consuming these fruits.

Materials and Methods

Plant Material

Mature *Berberis heteropoda* Schrenk fruits were collected from shrubs in the Nanshan Mountain area of Urumqi City, Xinjiang Uygur Autonomous Region, China (latitude 89°29′36″E, longitude 43°27′32″N), TH in September 2019. Fig. 1 shows its distribution in Xinjiang. The specimens were identified by expert Lude Xin from Xinjiang Medical University. A voucher specimen was deposited in the Institute of Clinical Nutrition, People's Hospital of Xinjiang Autonomous Region. High-quality *Berberis heteropoda* Schrenk fruit were selected, and stem and seeds were removed. The fruits were transported back to the laboratory for a pre-cooling treatment 2 h after harvest. Fruits with uniform size and maturity without mechanical damage were selected for dark storage at -20℃ until further analysis.

Fig. 1. *Berberis heteropoda* Schrenk shrub plant and distribution in Xinjiang.

Standards and Reagents

The reagents 1,1-diphenyl-2-picryl-hydrazl (DPPH) and 2,2-azinobis-(3-ethylbenzthiazoline-6- sulfonic acid) (ABTS) were purchased from Shanghai Macklin Biochemical Co., Ltd. Gallic acid and rutin standards were purchased from Chengdu Munster Biotechnology Co., Ltd. Folin–Ciocalteu’s phenol reagent was purchased from Tianjin Kaitong Chemical Reagent Co., Ltd. Anhydrous methanol, anhydrous ethanol,

concentrated hydrochloric acid, sodium nitrite, sodium hydroxide, sodium carbonate, and ferrous sulfate were obtained from Sinopharm Chemical Reagent Co., Ltd.

Nutritional Composition

Determination of general nutrients

The crude protein content was determined using the Kjeldahl method according to Chinese National Standard (CNS) GB/T5009.5-2016 “Determination of protein in food.” The ash content was measured using muffle furnace burning method according to CNS GB 5009.4-2016 “Determination of ash in food.” The crude fat was determined using the Soxhlet extraction method according to CNS GB 5009.6-20163 “Determination of fat in food.” The moisture was measured using the direct drying method according to CNS GB5009.3-2016 “Determination of moisture in food.” The carbohydrate content was determined based on CNS NY/T 2332-2013. The total energy of each sample was calculated as follows: Total Energy (kJ) = 17 × (g crude protein + g total carbohydrate) + 37 (g crude fat) [10].

Mineral composition

The mineral and element contents were determined according to CNS GB5009.268-2016 “Determination of multi-elements in food,” using inductively-coupled plasma-mass spectrometry (Agilent 5110 ICP-OES). Briefly, 1.0 g slurry sample was digested in 2 mL concentrated HNO_3 in a microwave oven and then diluted with distilled water to 25 mL. The solution was filtered before storage, and a blank digest was performed in a similar manner. The blank solution and the test solution were each injected into the ICP-OES , and the contents of K, Ca, Na, Mg, Fe, Cu, Zn, and P were determined.

Amino acid analysis

The amino acid contents were measured by an automatic amino acid analyzer (L-8900, Hitachi, Japan) according to the Chinese National Standard CNS GB 5009.124-2016 “Determination of amino acid in food.” Continuous flash evaporation at reduced pressure was used to remove excess acid, and the sample was dissolved in citrate buffer (pH 2.2) [11].

Fatty acids

The composition and content of fatty acids were determined by gas-chromatography mass spectroscopy (7890B/7000D Gas Chromatography-Mass Spectrometer, Agilent, USA) according to CNS GB 5009.168-2016. The triglyceride undecarbonate was applied as internal standard, and 37 different fatty acid methylester standard solutions were used for the external standard. The fatty acid content was quantitatively measured using the chromatographic peak.

Extraction and Quantification of Total Phenol ~~Content~~ (TPC), Total Flavonoid ~~Content~~ (TFC) and Total Anthocyanin Content (TAC) Content

Extraction

~~The samples were extracted using a modification method~~ [12]. Briefly, 1.0 g ~~stirred~~ *Berberis heteropoda* Schrenk fruit was obtained, 30 mL 70% acidified ethanol (0.1% v/v HCl) was added, and the solution was extracted three times under ultrasonic (40 kHz, 100 W) treatment for 30 min at 25 °C. Then, the mixture was centrifuged at 1000 r/min for 15 min, and the supernatant was collected. The residue was extracted twice, and all the collected supernatant was mixed, and then concentrated under vacuum. The extraction was preserved at -20°C until further analysis. This final extract was used for the quantification of TPC, TFC, TAC, and antioxidant activity.

Determination of TPC

The TPC was measured using Folin–Ciocalteu's phenol reagent with the colorimetric method [13]. Briefly, 0.5 mL of reagent and 1.5 mL of sodium carbonate solution (10% w/v) were added to 1 mL of *Berberis heteropoda* Schrenk fruit extract. Then, 8 mL pure water was immediately added, and the mixture was left for 10 min in a water bath at 75 ℃. Afterward, an ultraviolet–visible (UV–vis) spectrophotometer (PERSEE New century T6, China) was used to detect the absorbance at 760 nm. The standard curve of the absorbance value of gallic acid concentration solution was measured. TPC was denoted as milligram of gallic acid equivalent per gram of plant mass.

Determination of TFC

The TFC was measured using rutin as a reference standard with the aluminum nitrate method [14]. Briefly, 0.5 mL of *Berberis heteropoda* Schrenk fruit extract was added to 1 ml of sodium nitrite, left to stand for 6 min, mixed with 1 mL of 10% aluminum nitrate, and then left to stand again for 6 min. Afterward, 10 mL of 1.0 mol/L sodium hydroxide was added, the volume of water was fixed to 20 mL, and the solution was placed for 15 min. UV–vis spectrometry was used to detect the absorbance at 510 nm, and the standard curve of absorbance was determined. TFC was denoted as milligram of rutin equivalent per gram of weight of plant.

Determination of TAC

The TAC was determined by the pH differential method [15]. Briefly, 2 mL of fruit extract was added to the centrifuge tube and centrifuged at 1000 r/min for 5 min. Then, 0.5 mL supernatant was added into two equal 10 ml volumetric flasks: one with a pH 1.0 buffer, and the other with a pH 4.5 buffer. The absorbance at 517 nm and 700 nm was measured after 15

min balance. The data were expressed as milligram of cyanidin-3-glycoside equivalents per gram of fresh fruit weight. The TAC was calculated according to the following formula:

$$A = [(A_{517} - A_{700})_{pH1.0} - (A_{517} - A_{700})_{pH4.5}], \quad BHSTAC(mg/g) = A \times MW \times DF \times \frac{1}{\varepsilon \times L} \times \frac{V}{M},$$

where MW represents the molecular weight of centrinin-3-glycoside (449.2 g/mol), centrothrin-3- glycoside molar extinction coefficient (26900 L·cm^{-1}·mol^{-1}), DF represents diluted multiples, L denotes absorption pool thickness (1 cm), V represents extraction volume (mL), and M denotes the weight of peel powder].

Measurements of Antioxidant Capacity

DPPH free radical assay

The DPPH free radical scavenging assay was carried out according to the method described by Vlase et al [16]. Briefly, *Berberis heteropoda* Schrenk fruit extract was dissolved in 70% ethanol at different concentrations and mixed with 2 mL of a freshly prepared ethanol solution of DPPH free radicals (100 μmol/L). The solution was mixed vigorously and stored in darkness at room temperature for 30 min, and UV–vis spectrometry was used to detect the absorbance at 517 nm. The positive control group was measured with Vitamin C (V_C). The results were expressed as half maximal inhibitory concentration (IC50), which was used to indicate the corresponding concentration of the extract when the antioxygenation free radical scavenging capacity was 50%.

$$\text{DPPH free radical scavenging rate} = \left(1 - \frac{A_S - A_0}{A_C}\right) \times 100\%,$$

where A_C denotes absorbance value of the control, A_0 represents absorbance value of the blank, and A_S is the absorbance value of the sample.

ABTS free radical assay

The ABTS free radical scavenging assay was carried in accordance with the method described by Lyu et al [17]. Briefly, 2 mL of 10 mmol/L potassium persulfate solution and 2 mL of 10 mmol/L ABTS free radical solution were mixed and then stored in the dark for 12 h. Ethanol was added to the mixed solution until its UV–vis absorbance value reached 0.700 ± 0.020 at 736 nm. Subsequently, 2 mL of *Berberis heteropoda* Schrenk fruit extract or ascorbic acid solution was mixed vigorously with 2 mL of ABTS working solution and stored in the dark at room temperature for 10 min. The IC_{50} values of the sample extract were calculated based on the concentration and capacity of free radical scavenging curves.

$$\text{ABTS free radical scavenging rate} = \frac{A_C - A_S}{A_C} \times 100\%$$

where A_C represents absorbance value of the control and A_S denotes absorbance value of the sample.

Hydroxyl free radical assay

The hydroxyl free radical assay was carried out in accordance with the method described by Liang et al [18]. Briefly, 0.5 mL of 7.5 mmol/L ferrous sulfate heptahydrate, 0.5 mL of 7.5 mmol/L salicylic acid, 1 mL of *Berberis heteropoda* Schrenk fruit extract, and 0.2 mL of 30% hydrogen peroxide were mixed and left for 30 min in a water bath at 37 °C. After cooling, the absorbances of the sample, blank, and control groups were determined at 510 nm on the UV–vis spectrometer.

$$\text{HRSA}(\%) = \left(\frac{A_S - A_C}{A_0 - A_C}\right) \times 100\%;$$

where A_C denotes absorbance value of the control, A_0 represents absorbance value of the blank, and A_S represents absorbance value of the sample.

Superoxide anion free radical assay

The superoxide anion free radical assay was carried out in accordance with the method described by Liu et al [15]. Briefly, 4.5 mL of 50 mmol/L Tris-Hydrochloric acid and 1 mL of *Berberis heteropoda* Schrenk fruit extract were mixed and left for 15 min in a water bath at 25 °C. Afterward, 0.4 mL of 5 mmol/L pyrogallic acid was added and left for 5 min in a water bath at 25 °C. Subsequently, 0.1 mL of 8 mol/L hydrochloric acid was added to terminate the reaction, and the absorbance values of the sample, blank, and control were measured at 325 nm on the UV–vis spectrometer.

$$\text{Superoxide anion scavenging rate} = \frac{Ac - A_S}{A_S} \times 100\% ;$$

where Ac denotes absorbance value of the control, and as represents absorbance value of the sample.

Chromatography and Mass Spectrometry

Chromatographic conditions

Chromatographic separations were performed on an Agilent ultra-high performance liquid chromatography 1290 UPLC system with a Waters UPLC BEH C18 column (1.7 μm 2.1 × 100 mm). The flow rate was set to 0.4 mL/min, and the sample injection volume was set to 5 μL. The mobile phase consisted of 0.1% formic acid in water (A) and 0.1% formic acid in acetonitrile (B). The multi-step linear elution gradient program was as follows: 0–3.5 min, 95–85% A; 3.5–6 min, 85–70% A; 6–6.5 min, 70–70% A; 6.5–12 min, 70–30% A; 12–12.5 min, 30–30% A; 12.5–18 min, 30–0% A; 18–25 min, 0–0 % A; 25–26 min, 0–95% A; 26–30 min, 95–95% A.

Mass spectrometry (MS) conditions

An Q Exactive Focus mass spectrometer coupled with Xcalibur software was employed to obtain the MS and MS/MS data based on the IDA acquisition mode. During each acquisition cycle, the mass range was set from 100 to 1500，the top three of every cycle were screened, and the corresponding MS/MS data were further acquired. Sheath gas flow rate: 45 Arb; Aux gas flow rate: 15 Arb; Capillary temperature: 400 ℃; Full MS resolution: 70000; MS/MS resolution: 17500; Collision energy: 15/30/45 in NCE mode; Spray Voltage: 4.0 kV (positive) or -3.6 kV (negative).

Statistical Analysis

All experimental data were collected in triplicate, and the data were expressed as mean with standard deviation. Statistical analysis of the data was carried out in Graphpad Prism v7.0 (Graphpad Software, Inc.) and SPSS 23.0 (SPSS Inc., Chicago, IL, USA).

Results

Nutritional Composition of the Berberis heteropoda Schrenk Fruit

Proximate compositions of *Berberis heteropoda* Schrenk fruit

The major nutrients of *Berberis heteropoda* Schrenk fruit are summarized in Table 1. The major components were identified as water, crude fiber, and total protein. Water content was the highest (75.22±1.75 g/100 g), followed by crude fiber (17.72±0.52 g/100 g) and protein (2.55±0.03 g/100 g) contents. The ash content was 1.31±0.04 g/100 g, which indicates that the fruit is rich in minerals. The contents of total sugar and total fat were 0.05±0.00 and 0.51±0.02 g/100 g, respectively. The energy content per 100 g fruit was 363.52 kJ.

Table 1. Proximate compositions of *Breberis Heteropoda* Shrenk fruit

Compositions (Unit)	Data
Water (g/100 g fresh fruit)	75.22±1.75
Total fat (g/100 g fresh fruit)	0.506±0.02
Total Protein (g/100 g fresh fruit)	2.55±0.03
Ash (g/100 g fresh fruit)	1.31±0.04
Total sugars (g/100 g fresh fruit)	0.05±0.00
Carbohydrates (g/100 g fresh weight)	17.72±0.52
Total Energy (kJ)	363.52±7.51

Minerals

A total of eight mineral elements were detected in *Berberis heteropoda* Schrenk fruit and are summarized in Table 2. Potassium (582.67±8.02 mg/100 g) was the most abundant element [19]. Calcium (78.5±1.62 mg/100 g), phosphorus (73.24±1.72 mg/100 g), and magnesium (30.61±0.56 mg/100 g) were also abundant.

Table 2. Nutritional composition (minerals, amino acids) of *Breberis Heteropoda* Shrenk fruit

Minerals	mg Per 100 g of FW	Percentage of Total Minerals (%)
Na	1.38±0.03	0.18
K	582.67±8.02	75.73
Ca	78.5±1.62	10.20
Cu	0.27±0.01	0.04
Zn	0.59±0.01	0.08
Fe	2.31±0.05	0.30

Mg	30.61±0.56	3.97
P	73.24±1.72	9.51
Total	769.843	

**Amino Acids**	**g Per 100 g of FW**	**Percentage of Total Amino Acids (%)**
Phenylalanine	0.12±0.01	4.24
Alanine	0.17±0.01	6.01
Methionine	0.015±0.00	0.53
proline	0.2±0.02	7.07
glycine	0.21±0.01	7.43
Glutamic acid	0.53±0.01	18.74
arginine	0.22±0.01	7.78
lysine	0.21±0.00	7.43
tyrosine	0.11±0.00	3.89
Leucine	0.18±0.01	6.36
Serine	0.13±0.01	4.60
threonine	0.13±0.01	4.60
Aspartic acid	0.27±0.01	9.55
valine	0.15±0.01	5.30
histidine	0.073±0.00	2.58
isoleucine	0.11±0.01	3.89
Total amino acids	2.828	

Amino acids

The details of 16 identified amino acids in *Berberis heteropoda* Schrenk fruit are shown in Table 2. Glutamic acid was the most abundant, and the contents of aspartic acid, arginine, lysine, and glycine were also abundant. It contained 6 types of essential amino acids (EAAs), and the content of essential amino acids was 0.9 g/100 g, accounting for 31.8% of the total amino acids. The content of remaining ten non-EAAs (NEAAs) was 1.93 g/100 g.

Fatty acids

The content of fatty acid in *Berberis heteropoda* Schrenk fruit is presented in Table 3. A total of 10 different fatty acids were determined, including saturated and unsaturated varieties. Tetrahexanoic acid (C24:0) was found to be the dominant fatty acid, followed by octadecentrienoic acid (C18:3) and octadecadienoic acid (C18:2). Unsaturated fatty acid content (UFA, 51.52%) was slightly higher than that of saturated fatty acids (SFA, 48.48%).

Table 3. Fatty Acid Content in *Breberis Heteropoda* Shrenk fruit

Fatty acids	Formula	g/100 g fatty acid	Proportion (%)
Myristic acid (C14:0)	$C_{14}H_{28}O_2$	0.0039	1.41
2-methyl-heptanoic acid (C8:0)	$C_8H_{16}O_2$	0.0019	0.71
Hexadecanoic acid (C16:0)	$C_{16}H_{32}O_2$	0.0285	10.35
Stearyl acid (C18:0)	$C_{18}H_{36}O_2$	0.0041	1.50
Octadecenoic acid (C18:1)*	$C_{18}H_{32}O_2$	0.0263	9.55
Octadecadienoic acid (C18:2)*	$C_{18}H_{32}O_2$	0.0526	19.11
Octadecentrienoic acid (C18:3)*	$C_{18}H_{30}O_2$	0.0630	22.86
Arachidic acid (C20:0)	$C_{20}H_{40}O_2$	0.0038	1.38

Docosanoic acid (C22:0)	$CH_3(CH2)_{20}COOH$	0.0112	4.06
Tetrahexanoic acid (C24:0)	$CH_3(CH2)_{22}COOH$	0.0801	29.08
Subtotal		0.2754	100.00

Note: *unsaturated fatty acida

TPC, TFC, TAC, and Antioxidant Activity of Berberis heteropoda Schrenk Fruit Extract

The TPC, TFC, and TAC values in *Berberis heteropoda* Schrenk fruit are shown in Fig. 2. The determination methods of flavonoids and polyphenols exhibited a good linear relationship within the measurement range ($r^2 = 0.995$; $r^2 = 0.999$). The regression equations used were $y = 0.0109x + 0.0157$ and $y = 0.067x - 0.0173$. Compared with the extraction effect of different solvents, the extraction effect of solvent from high to low is methanol > acetone > ethanol for total phenol, total flavonoids, and total anthocyanins. Using methanol as extraction solvent, the TFC, TPC, and TAC values were 108.42, 68.55, and 19.83 mg/g fruit, respectively. Evidently, with methanol as the extraction solvent, total flavonoids and total phenol can obtain a higher extraction rate.

Fig. 2. Quantification of TPC (A), TFC (B), TAC (C) of *Berberis heteropoda* Schrenk shrub extract.

The antioxidant activity of the *Berberis heteropoda* Schrenk fruit extracts was evaluated using Vc as the control, and the IC_{50} value of DPPH free radical, ABTS, •OH, O_2•- scavenging ability were 20.27±0.26 μg/mL, 13.89±0.13 mg/mL, 5.81±0.13 mg/mL, and 0.57±0.02 mg/mL, respectively (Fig. 3). We observed that methanol extract had the best antioxidant activity, and the IC_{50} of DPPH radical, hydroxyl radical, ABTS radical, and superoxide anion radical were 20.13 μg/mL, 5.44 mg/mL, 8.79 μg/mL, and 1.35 mg/ mL, respectively. The IC_{50} values of methanol extraction were higher than those of vitamin C but lower than those of

ethanol and acetone extraction. Methanol, acetone, and ethanol extracts were ranked from high to low in the order of free radical scavenging ability, and *Berberis heteropoda* Schrenk fruit extracts had good antioxidant activity and could scavenge free radicals effectively.

Fig. 3. IC_{50} value (mg/mL) of different extracts on radicals

(A: DPPH radical; B: ABTS radical; C: hydroxyl radical; D: superoxide anion radical).

Identification of Phenols in Berberis heteropoda Schrenk Fruit Extract

The UPLC-QTOF-MS spectra indicated that the compounds in the extract of *Berberis heteropoda* Schrenk fruit were primarily concentrated within 2–10 min, when the mobile phase was 15%–70% ethyl alcohol solution, indicating that the polyphenols of *Berberis heteropoda* Schrenk fruit belonged to polar compounds (Table 4; Fig. 4). The details of 32 kinds of ~~compounds are listed as follows~~: Compounds 1 (t_R=1.52 min), 2 (t_R=2.96 min), and 3 (t_R=3.67 min) with fragment $[M-H]^-$ ions at m/z 633.0787 [12], 477.1030 [20], and 289.0719 [21]. Compounds 4 (t_R=4.53 min), 5 (t_R=5.03 min), and 6 (t_R = 5.50 min) with fragment $[M-H]^-$ ions at m/z 479.0835 [22], 507.1144 [23], and 479.0834 [24]. Compounds 7 (t_R = 5.80 min), 8 (t_R = 5.84 min), and 9 (t_R =5.85 min) with fragment $[M-H]^-$ ions at m/z 447.0926, 507.1143, and 449.1095 [24,25]. Compounds 10 (t_R=5.89 min), 11 (t_R = 5.91 min), and 12 (t_R = 6.02 min) with fragment $[M-H]^-$ ions at m/z 285.0393, 463.0892, and 317.0300 [10,24]. Compounds 13 (t_R = 6.14 min), 14 (t_R=6.20 min), and 15 (t_R =6.73 min) with fragment $[M-H]^-$ ions at m/z 447.0927 [24], 319.0458 [26], and 301.0357 [27]. Compounds 16 (t_R=6.97 min), 17 (t_R=7.97 min), and 18 (t_R=8.04 min) with fragment $[M-H]^-$ ions at m/z 301.0358, 285.0408, and 299.0556 [28,29]. Compounds 19 (t_R=8.19 min), 20 (t_R=9.56 min), and 21 (t_R=4.52 min) with fragment $[M-H]^-$ ions at m/z 315.0507 [21], 269.0455 [29] and fragment $[M+H]^+$ ions at m/z 271.0588 [30]. Compounds 22 (t_R=4.75 min), 23 (t_R=4.85 min), and 24 (t_R=5.05 min) with fragment $[M+H]^+$ ions at m/z 285.1122 [31], 291.0858 [32], and 303.0478 [33]. Compounds 25 (t_R=5.08 min), 26

(t_R=5.55 min), and 27 (t_R = 7.25 min) with fragment $[M+H]^+$ ions at m/z 305.0650, 333.0602, and 435.127884 [24,34]. Compounds 28 (t_R=7.29 min), 29 (t_R=7.75 min), and 30 (t_R=8.22 min) with fragment $[M+H]^+$ ions at m/z 437.1445 [35], 437.1445 [36], and 437.1445 [36]. Finally, compounds 31 (t_R=9.92 min) and 32 (t_R=14.88 min) with fragment $[M+H]^+$ ions at m/z 437.1445 and 595.1658 [10,38].

Fig. 4. Representative total ion chromatogram of extract sample obtained from Berberis heteropoda Schrenk fruit in positive mode.

(A) and in negative mode (B) by UHPLC-ESI-Q-TOF/MS.

Table 4. Characterisation of phenolic compounds of *Breberis Heteropoda* Shrenk fruit by UPLC-Q-TOF-MSE.

Compound	t_R/min	Ionisation mode	Identification	Molecular formula	MS(m/z)	MS^2(m/z)
1	1.52	$[M-H]^-$	Corilagin	C27H22O18	633.0787	261.667; 181.051
2	2.96	$[M-H]^-$	Petunidin-3-O-beta-glucopyranoside	C22H23O12	477.1030	299.013; 314.043
3	3.67	$[M-H]^-$	Cianidanol	C15H14O6	289.0719	245.0827
4	4.53	$[M-H]^-$	Gossypetin-8-C-glucoside	C21H20O13	479.0835	316.0244; 271.216
5	5.03	$[M-H]^-$	Syringetin-3-O-glucoside	C23H24O13	507.1144	301.067; 345.0604
6	5.50	$[M-H]^-$	Myricetin-3-O-galactoside	C21H20O13	479.0834	115.0551; 133.014
7	5.80	$[M-H]^-$	kaempferol 7-O-glucoside	C21H20O11	447.0926	285.0365
8	5.84	$[M-H]^-$	Syringetin-3-O-galactoside	C23H24O13	507.1143	344.053; 273.032
9	5.85	$[M-H]^-$	Flavanomarein	C21H22O11	449.1095	287.0572; 150.0037
10	5.89	$[M-H]^-$	Luteolin	C15H10O6	285.0393	151.0022; 133.0302
11	5.91	$[M-H]^-$	Spiraeoside	C21H20O12	463.089	301.034; 179.0188
12	6.02	$[M-H]^-$	Myricetin	C15H10O8	317.0300	137.0248; 151.00568

13	6.14	$[M-H]^-$	Luteolin-4'-O-glucoside	C21H20O11	447.0927	285.0388
14	6.20	$[M-H]^-$	Dihydromyricetin	C15H12O8	319.0458	150.999; 107.0111
15	6.73	$[M-H]^-$	Morin	C15H10O7	301.0356	165.02
16	6.97	$[M-H]^-$	Quercetin	C15H10O7	301.0357	121.0272; 151.0038; 178.9974
17	7.97	$[M-H]^-$	Kaempferol	C15H10O6	285.0408	
18	8.04	$[M-H]^-$	Kaempferide	C16H12O6	299.0556	284.0329; 256.036
19	8.19	$[M-H]^-$	Isorhamnetin	C16H12O7	315.0507	300.029
20	9.56	$[M-H]^-$	Galangin	C15H10O5	269.0455	225.0558
21	4.52	$[M+H]^+$	Genistein	C15H10O5	271.0588	121.028
22	4.75	$[M+H]^+$	Flavokawain B	C17H16O4	285.1122	249.1829; 267.141
23	4.85	$[M+H]^+$	Epicatechin	C15H14O6	291.0858	123.0446; 139.039
24	5.05	$[M+H]^+$	Herbacetin	C15H10O7	303.0478	257.042
25	5.08	$[M+H]^+$	Dihydro-Quer	C15H12O7	305.0650	289.631; 290.365
26	5.55	$[M+H]^+$	Flavonol base + 4O, 1MeO	C16H12O8	333.0602	58.065; 318.036
27	7.25	$[M+H]^+$	Naringenin-7-O-glucoside	C21H22O10	435.1279	153.0385; 273.0744
28	7.29	$[M+H]^+$	Phlorizin	C21H24O10	437.1445	107.045; 275.0905
29	7.75	$[M+H]^+$	Hyperoside	C21H20O12	465.1028	61.0285; 85.0285
30	8.22	$[M+H]^+$	Aurantio-obtusin beta-D-glucoside	C23H24O12	493.1329	331.0826
31	9.92	$[M+H]^+$	Kaempferol 3-glucorhamnoside	C27H30O15	595.1650	85.0305; 287.0686
32	9.92	$[M+H]^+$	Vicenin 2	C27H30O15	595.1658	325.071; 317.0645

Discussion

Berberis heteropoda Schrenk is an important local plant ~~resource~~ in the Nanshan Mountain region, and understanding the active components of the plant can guide its nutritional utilization. A prior study on anthocyanin composition in *Berberis heteropoda* Schrenk fruit considered it as a potential anthocyanin pigment source [3]. This study focused on the chemical information of *Berberis heteropoda* Schrenk fruit; however, there has been a lack of comprehensive scientific research on the overall nutritional composition of the fruit. This study systematically evaluated the major nutrients and antioxidant properties of *Berberis heteropoda* Schrenk fruits and found them to be rich in various nutrients, providing evidence for potential healthcare or nutritional use. Moreover, a total of 32 ~~kinds of~~ polyphenols were detected from *Berberis heteropoda* Schrenk fruit extract.

This study revealed that *Berberis heteropoda* Schrenk fruit exhibited nutritional properties suggesting potential nutraceutical value. Moreover, the major nutrients of *Berberis heteropoda* Schrenk fruit was comparable to that of wolfberry (*Lycium ruthenicum* Murr), which as a wild plant and widely observed in Xinjiang [39]. Moreover, the protein content in *Berberis heteropoda* Schrenk fruit was higher than black mulberry (1.17±0.06%) [19]. Furthermore, the content of fat and sugar in *Berberis heteropoda* Schrenk fruit was lower, which did not cause excess risk of obesity, and it could be considered a functional food or medicine rather than edible fresh fruits due to poor taste.

Our study revealed that *Berberis heteropoda* Schrenk fruit contained a large amount of minerals, including sodium, potassium, calcium, copper, zinc, iron, magnesium, phosphorus, and other elements. Studies have demonstrated that such minerals play an important role in the physiological function of human tissues, maintaining the osmotic pressure of cells, supporting the pH balance of the body, and regulating special physiological functions of the body as cofactors [40,41]. Moreover, we observed that sodium/potassium ratio in *Berberis heteropoda* Schrenk fruit was 0.002, which could prevent hypertension risk [42]. Based on

these findings, *Berberis heteropoda* Schrenk fruit may be considered helpful for controlling blood pressure.

The ratio of EAAs to NEAAs was 0.47, which does not meet the ideal protein condition proposed by FAO/WHO [43], and therefore, this fruit is not recommended as a high-quality protein food. The percentage of glutamic acid, glycine, and aspartic acid were 18.72, 7.42, and 9.54 %, respectively, accounting for more than one-third of the total amino acids in *Berberis heteropoda* Schrenk fruit. Moreover, the UFA: SFA ratio in *Berberis heteropoda* Schrenk fruit was 1.06, suggesting that *Berberis heteropoda* Schrenk fruit should not be recommended as a rich fatty acid food.

Phenolic and flavonoid compounds have been demonstrated as important phytonutrients ~~in plants~~ [44,45]. Flavonoids are the secondary metabolites with activity in plants, which are widely existed and abundant [46]. Phenols are important plant compounds that mimic the biological effects of vitamin E [47]. Moreover, a previous study revealed that anthocyanins are rich in many plants, and the fruits presented red, yellow, purple, black, and other ~~colorful~~ colors induced by anthocyanins [48]. The basic structural unit of anthocyanins is 2-phenylbenzopyran, comprising C6-C3-C6 skeleton [49]. Because of its unique structure, it has the functions of anti-oxidation, anti-inflammation, anti-tumor [50], prevention of cardiovascular disease, and enhancement of vision [51]. Our study found that the content of *Berberis heteropoda* Schrenk fruit was higher than that of wolfberry [39] but lower than that of *Passiflora foetida* [10]. This result suggested that TPC is significantly related to geographical, climate, and soil conditions. The anthocyanin content of *Berberis heteropoda* Schrenk fruit found in this study was slightly inconsistent with a prior study, possibly because the previous samples were obtained from Yili, Xinjiang [3].

The free radical scavenging activity of flavonoids and polyphenols has been demonstrated in previous study [52]. The IC_{50} value is typically used to evaluate the antioxidant activity, and

smaller IC_{50} values indicate stronger antioxidant capacity. The present results indicated that *Berberis heteropoda* Schrenk fruit extract had strong scavenging effects on DPPH•, •OH, O_2•- and $ABTS^+$•. These results suggested that *Berberis heteropoda* Schrenk fruit could be considered an excellent source of natural antioxidants.

This study provides an examination of the major nutrients and antioxidant properties of *Berberis heteropoda* Schrenk fruits. However, several shortcomings should be mentioned: (1) functional monomers of the *Berberis heteropoda* Schrenk fruits were not addressed; (2) structures of specific phenolic compounds and their antioxidant effects were not assessed, and the mechanism of action needs further evaluation; and (3) potential effect of *Berberis heteropoda* Schrenk fruits on health needs further assessment.

Conclusions

The present study analyzed the major nutrients, mineral elements, fatty acids, and amino acids of *Berberis heteropoda* Schrenk fruits and found a wide array of important nutrient components. Moreover, the TPC, TFC, and TAC values were high, with excellent antioxidant properties. Therefore, *Berberis heteropoda* Schrenk fruit may be used as a potential health-promoting food for resisting oxidation damage.

Financial Disclosure Statement

This research was funded by the Research Foundation of Xinjiang Uygur Autonomous Region (Natural Science Foundation) Joint Fund Project, grant number 2021D01C177

Competing interests

The authors declare no conflict of interest.

Acknowledgments. The authors would like to thank expert Lude Xin for identifying the plants, Guangzhou Jinzhi Testing Technology Co., Ltd for excellent technical assistance during this study, and Professor Yu Wei from Dalian Institute of Chemical Physics, Chinese Academy of Sciences for editorial assistance with the manuscript.

Data availability statement. The datasets generated and/or analysed during the current study are available from the corresponding author on reasonable request.

Author Contributions. Conceptualization: JGL; Methodology: JXS and QL; Writing-original draft preparation: JXS and FX; Writing-review and editing: JXS; Data curation: FX and JL; Project administration: JGL.

References

1. Zhu HM, Li L, Li SY, *et al*. Effect of water extract from Berberis heteropoda Schrenk roots on diarrhea-predominant irritable bowel syndrome by adjusting intestinal flora. *J Ethnopharmacol* 2019; 237:182–191.

2. Xu X, Baharguli H, editors. Kazak Medicine Blog. 1st ed. Volume 1. The Ethnic Publishing House; Beijing, China: 2009. p. 156.

3. Sun LL, Gao W, Zhang MM, *et al.* Composition and antioxidant activity of the anthocyanins of the fruit of Berberis heteropoda Schrenk. *Molecules* 2014;19:19078-19096.

4. Ministry of Health of Forces Logistics of Xinjiang. Handbook of Chinese Herbal of Xinjiang. People’s Publishing House; Xinjiang, China: 1970. pp. 153–283.

5. Li L, Zhu HM, Yan Q, *et al*. The antibacterial activity of Berberis heteropoda Schrenk and its effect on irritable bowel syndrome in rats. *Chin J Nat Med* 2020;18:356-368.

6. De Marino S, Festa C, Zollo F, *et al*. Antioxidant activity and chemical components as potential anticancer agents in the olive leaf (Olea europaea L. cv Leccino.) decoction. *Anticancer Agents Med Chem* 2014;14:1376-85.

7. Alara OR, Abdurahman NH, Ukaegbu CI. Extraction of phenolic compounds: A review. *Curr Res Food Sci* 2021;4:200-214.

8. Cianciosi D, Forbes-Hernández TY, Afrin S, *et al.* Phenolic Compounds in Honey and Their Associated Health Benefits: A Review. *Molecules* 2018;23:2322.

9. Abdullah NA, Zullkiflee N, Zaini S, *et al.* Phytochemicals, mineral contents, antioxidants, and antimicrobial activities of propolis produced by Brunei stingless bees Geniotrigona thoracica, Heterotrigona itama, and Tetrigona binghami. *Saudi J Biol Sci* 2020;27:2902-2911.

10. Song Y, Wei XQ, Li MY, *et al.* Nutritional Composition and Antioxidant Properties of the Fruits of a Chinese Wild Passiflora foetida. *Molecules* 2018;23:459.

11. Darragh AJ, Moughan PJ. The effect of hydrolysis time on amino acid analysis. *J AOAC Int* 2005; 88:888-93.

12. Liu Z, Zeng Y, Hou P. Metabolomic evaluation of Euphorbia pekinensis induced nephrotoxicity in rats. *Pharm Biol* 2018;56:145-153.

13. Wei F, Chen Q, Du Y, *et al.* Effects of hulling methods on the odor, taste, nutritional compounds, and antioxidant activity of walnut fruit. *LWT* 2020;120:108938.

14. Yang L, Liang Q, Zhang Y, *et al.* Variation of phytochemical composition of Lycium chinense leaves as an endemic high-value healthy resource. *Scientia Horticulturae* 2021; 281:109910.

15. Liu Z, Tang X, Liu C, *et al.* Ultrasonic extraction of anthocyanins from Lycium ruthenicum Murr. and its antioxidant activity. *Food Sci Nutr* 2020;8:2642-2651.

16. Zhu M, Zhao H, Wang Q, *et al.* A Novel Chinese Honey from Amorpha fruticosa L.: Nutritional Composition and Antioxidant Capacity In Vitro. *Molecules* 2020;25:5211.

17. Lyu Y, Bi J, Chen Q, *et al.* Bioaccessibility of carotenoids and antioxidant capacity of seed-used pumpkin byproducts powders as affected by particle size and corn oil during in vitro digestion process. *Food Chem* 2021;343:128541.

18. Liang L, Wang C, Li S, *et al.* Nutritional compositions of Indian Moringa oleifera seed and antioxidant activity of its polypeptides. *Food Sci Nutr* 2019;7:1754-1760.

19. Jiang Y, Nie WJ. Chemical properties in fruits of mulberry species from the Xinjiang province of China. *Food Chem* 2015;174:460-6.

20. Brito A, Areche C, Sepúlveda B, *et al.* Anthocyanin characterization, total phenolic quantification and antioxidant features of some Chilean edible berry extracts. *Molecules* 2014;19:10936-10955.

21. Zhou J, Cai H, Tu S, *et al.* Identification and Analysis of Compound Profiles of Sinisan Based on 'Individual Herb, Herb-Pair, Herbal Formula' before and after Processing Using UHPLC-Q-TOF/MS Coupled with Multiple Statistical Strategy. *Molecules* 2018;23:3128.

22. Wang H, Wu R, Xie D, *et al.* A Combined Phytochemistry and Network Pharmacology Approach to Reveal the Effective Substances and Mechanisms of Wei-Fu-Chun Tablet in the Treatment of Precancerous Lesions of Gastric Cancer. *Front Pharmacol* 2020;11:558471.

23. Yang M, Wang L, Belwal T, *et al*. Exogenous Melatonin and Abscisic Acid Expedite the Flavonoids Biosynthesis in Grape Berry of Vitis vinifera cv. Kyoho. *Molecules* 2019;25:12.

24. Tan L, Jin Z, Ge Y, *et al*. Comprehensive ESI-Q TRAP-MS/MS based characterization of metabolome of two mango (Mangifera indica L) cultivars from China. *Sci Rep* 2020;10:20017.

25. Yang Y, Sun X, Liu J, *et al*. Quantitative and Qualitative Analysis of Flavonoids and Phenolic Acids in Snow Chrysanthemum (Coreopsis tinctoria Nutt.) by HPLC-DAD and UPLC-ESI-QTOF-MS. *Molecules* 2016;21:1307.

26. Cádiz-Gurrea ML, Lozano-Sánchez J, Fernández-Ochoa Á, *et al.* Enhancing the Yield of Bioactive Compounds from Sclerocarya birrea Bark by Green Extraction Approaches. *Molecules* 2019;24: 966.

27. Wali AF, Pillai JR, Al Dhaheri Y, *et al.* Crocus sativus L. Extract Containing Polyphenols Modulates Oxidative Stress and Inflammatory Response against Anti-Tuberculosis Drugs-Induced Liver Injury. *Plants (Basel)* 2020;9:167.

28. Yang R, Guan Y, Zhou J, *et al.* Phytochemicals from Camellia nitidissima Chi Flowers Reduce the Pyocyanin Production and Motility of Pseudomonas aeruginosa PAO1. *Front Microbiol* 2018;8: 2640.

29. Xu X, Yang B, Wang D, *et al.* The Chemical Composition of Brazilian Green Propolis and Its Protective Effects on Mouse Aortic Endothelial Cells against Inflammatory Injury. *Molecules* 2020;25:4612.

30. Liu JL, Li LY, He GH. Optimization of Microwave-Assisted Extraction Conditions for Five Major Bioactive Compounds from Flos Sophorae Immaturus (Cultivars of Sophora japonica L.) Using Response Surface Methodology. *Molecules* 2016;21:296.

31. Tang Y, Fields C. A UHPLC-UV Method Development and Validation for Determining Kavalactones and Flavokavains in Piper methysticum (Kava). *Molecules* 2019;24:1245.

32. Shoko T, Maharaj VJ, Naidoo D, *et al.* Anti-aging potential of extracts from Sclerocarya birrea (A. Rich.) Hochst and its chemical profiling by UPLC-Q-TOF-MS. *BMC Complement Altern Med* 2018;18:54.

33. Kramberger K, Barlič-Maganja D, Bandelj D, *et al.* HPLC-DAD-ESI-QTOF-MS Determination of Bioactive Compounds and Antioxidant Activity Comparison of the Hydroalcoholic and Water Extracts from Two Helichrysum italicum Species. *Metabolites* 2020;10:403.

34. Islam S, Alam MB, Ann HJ, *et al.* Metabolite Profiling of Manilkara zapota L. Leaves by High-Resolution Mass Spectrometry Coupled with ESI and APCI and In Vitro Antioxidant Activity, α-Glucosidase, and Elastase Inhibition Assays. *Int J Mol Sci* 2020;22:132.

35. Ma Z, Huang Y, Huang W, *et al.* Separation, Identification, and Antioxidant Activity of Polyphenols from Lotus Seed Epicarp. *Molecules* 2019;24:4007.

36. Dai L, He J, Miao X, *et al.* Multiple Biological Activities of Rhododendron przewalskii Maxim. Extracts and UPLC-ESI-Q-TOF/MS Characterization of Their Phytochemical Composition. *Front Pharmacol* 2021;12:599778.

37. Song W, Jiang W, Wang C, *et al.* Jinmaitong, a Traditional Chinese Compound Prescription, Ameliorates the Streptozocin-Induced Diabetic Peripheral Neuropathy Rats by Increasing Sciatic Nerve IGF-1 and IGF-1R Expression. *Front Pharmacol* 2019;10:255.

38. Yang L, Liu RH, He JW. Rapid Analysis of the Chemical Compositions in Semiliquidambar cathayensis Roots by Ultra High-Performance Liquid Chromatography and Quadrupole Time-of-Flight Tandem Mass Spectrometry. *Molecules* 2019;24:4098.

39. Liu Z, Liu B, Wen H, *et al.* Phytochemical profiles, nutritional constituents and antioxidant activity of black wolfberry (Lycium ruthenicum Murr.). *Industrial Crops and Products* 2020;154:112692.

40. Katarzyna J, Karolina J, Patrycja K, *et al.* Mineral Composition and Antioxidant Potential in the Common Poppy (Papaver rhoeas L.) Petal Infusions. *Biol Trace Elem Res* 2021;199:371-381.

41. Lenzi A, Orlandini A, Bulgari R, *et al.* Antioxidant and Mineral Composition of Three Wild Leafy Species: A Comparison Between Microgreens and Baby Greens. *Foods* 2019;8:487.

42. Xu J, Chen X, Ge Z, *et al.* Associations of Usual 24-Hour Sodium and Potassium Intakes with Blood Pressure and Risk of Hypertension among Adults in China's Shandong and Jiangsu Provinces. *Kidney Blood Press Res* 2017;42:188-200.

43. Ramu R, Shirahatti PS, Anilakumar KR, *et al.* Assessment of Nutritional Quality and Global Antioxidant Response of Banana (Musa sp. CV. Nanjangud Rasa Bale) Pseudostem and Flower. *Pharmacognosy Res* 2017;9:S74-S83.

44. Stinco CM, Sentandreu E, Mapelli-Brahm P, *et al.* Influence of high pressure homogenization and pasteurization on the in vitro bioaccessibility of carotenoids and flavonoids in orange juice. *Food Chem* 2020;331:127259.

45. Behl T, Kumar K, Brisc C, *et al.* Exploring the multifocal role of phytochemicals as immunomodulators. *Biomed Pharmacother* 2021;133:110959.

46. Khalid M, Saeed-ur-Rahman, Bilal M, *et al.* Role of flavonoids in plant interactions with the environment and against human pathogens-A review. *J Integrat Agricul* 2019;18:211-230.

47. Peanparkdee M, Patrawart J, Iwamoto S. Physicochemical stability and in vitro bioaccessibility of phenolic compounds and anthocyanins from Thai rice bran extracts. *Food Chem* 2020;329:127157.

48. Chen J, Du J, Li M, *et al.* Degradation kinetics and pathways of red raspberry anthocyanins in model and juice systems and their correlation with color and antioxidant changes during storage. *LWT* 2020; 128:109448.

49. Yao L, Xu J, Zhang L, *et al.* Nanoencapsulation of anthocyanin by an amphiphilic peptide for stability enhancement. Food Hydrocolloids 2021;118:106741.

50. Zhang H, Xu Z, Zhao H, *et al.* Anthocyanin supplementation improves anti-oxidative and anti-inflammatory capacity in a dose-response manner in subjects with dyslipidemia. *Redox Biol* 2020; 32:101474.

51. Mehmood A, Zhao L, Wang Y, *et al.* Dietary anthocyanins as potential natural modulators for the prevention and treatment of non-alcoholic fatty liver disease: A comprehensive review. *Food Res Int* 2021;142:110180.

52. Guo H, Saravanakumar K, Wang M. Total phenolic, flavonoid contents and free radical scavenging capacity of extracts from tubers of Stachys affinis. Biocatal Agricul Biotech 2018; 15:235-239.

Fig. 1

Click here to access/download;Figure;Fig. 1.tif

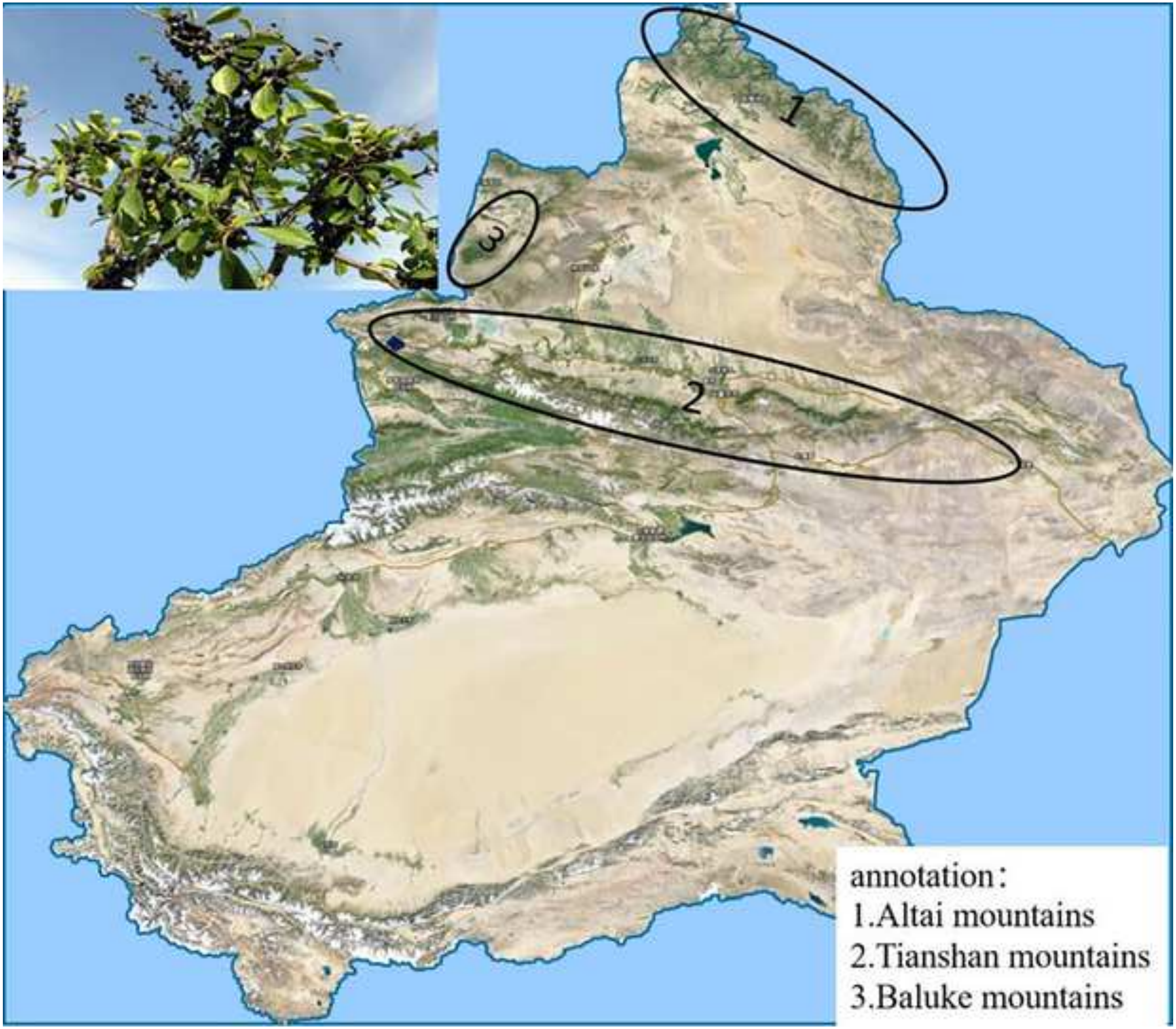

Fig. 2

Click here to access/download;Figure;Fig. 2.tif

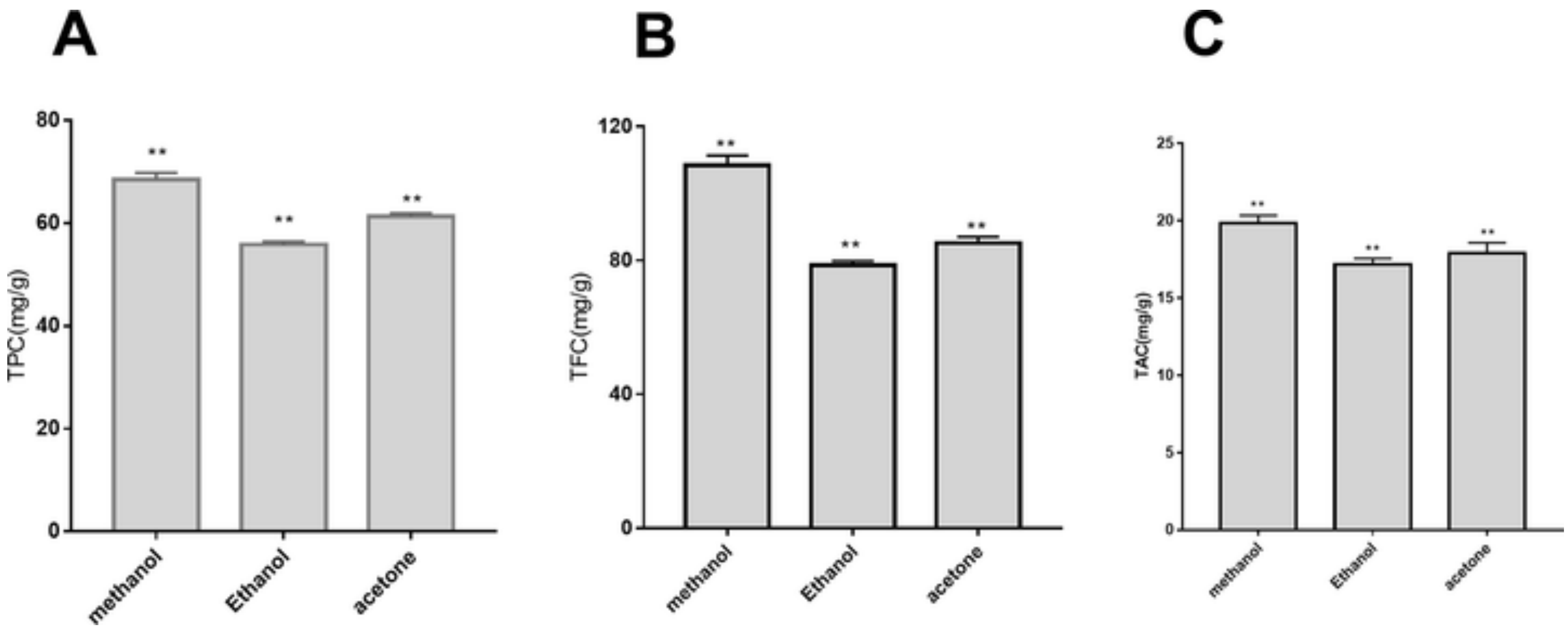

Fig. 3

Click here to access/download;Figure;Fig. 3.tif

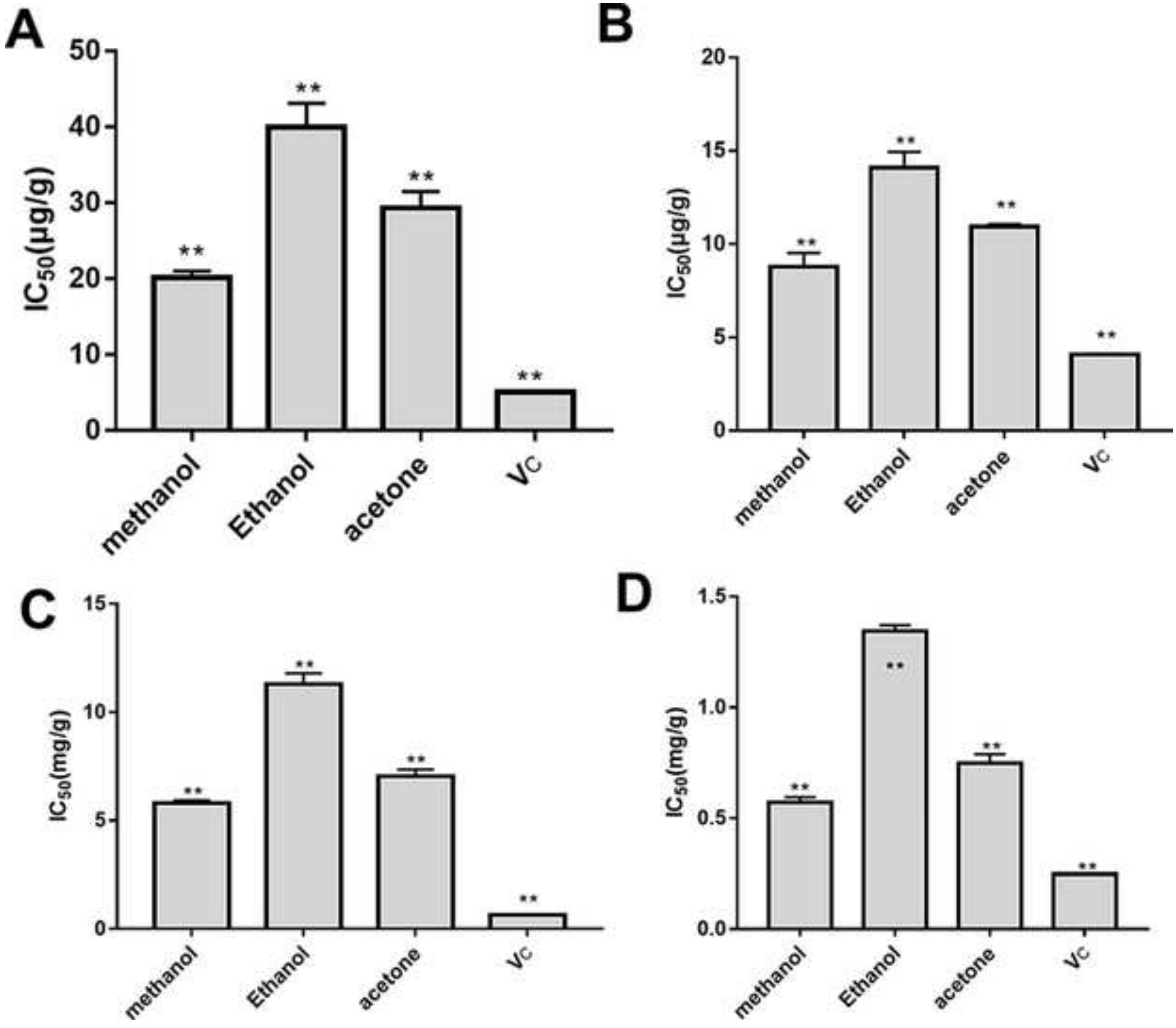

Fig. 4

Click here to access/download;Figure;Fig. 4.pdf

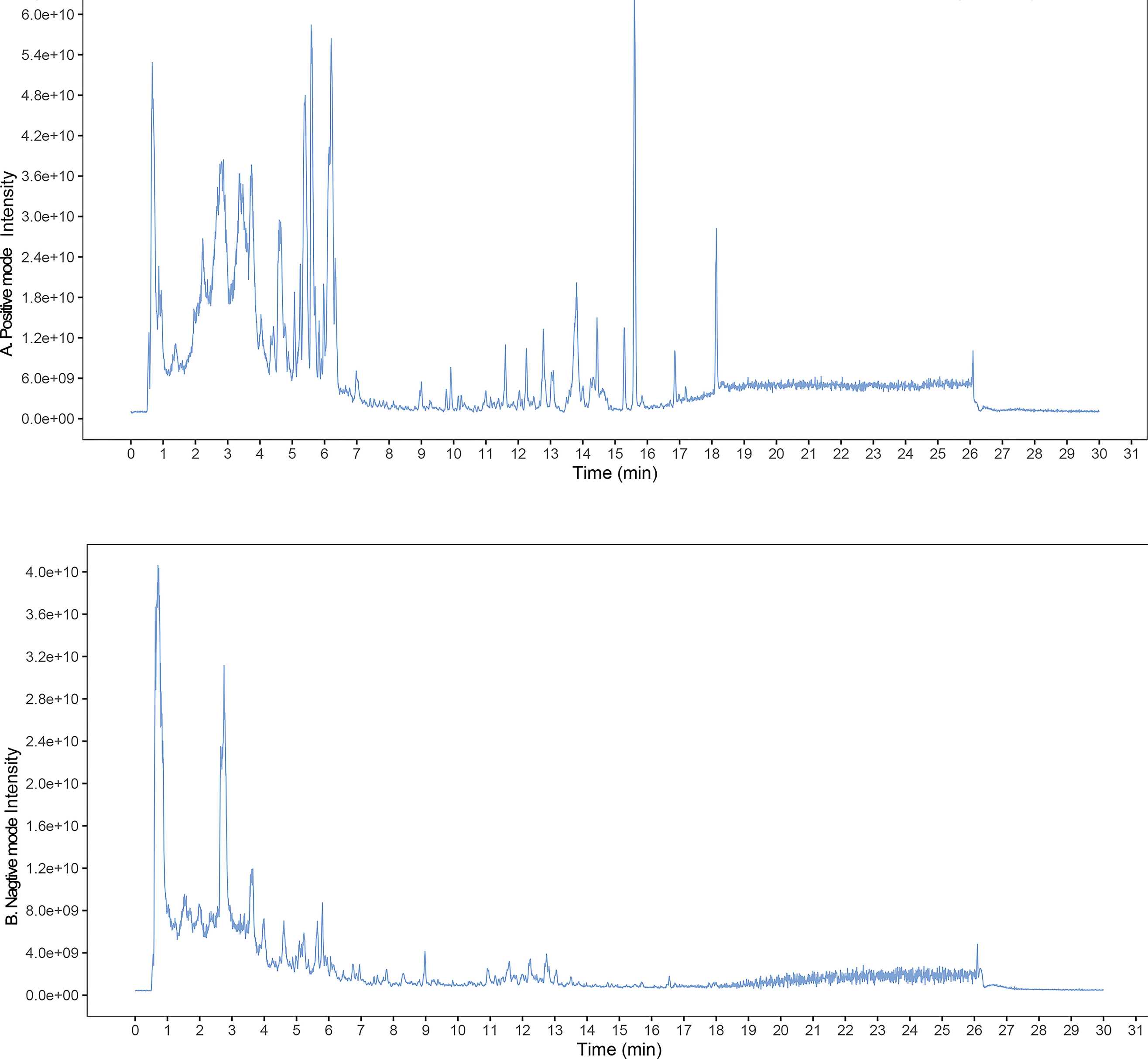

Supplement: Attachment — Submitted filename: PONE-D-21-29715_reviewer.pdf [file pone.0262622.s005.pdf]
