# Supplementary material for: Nutritional composition and antioxidant properties of the fruit of Berberis heteropoda Schrenk
Source: PLoS One. 2022 Apr 7;17(4):e0262622. doi: 10.1371/journal.pone.0262622 (PMC8989241; doi:10.1371/journal.pone.0262622)
Supplement: Attachment — Submitted filename: Response to reviewer.docx [file pone.0262622.s007.docx]

**Point-By-Point Response**

**Response to editor**

General comments: The authors need to thoroughly reorganize the Discussion section, since some Reviewers' concerns were not properly addressed. Up-to-date literature that deals with the same topic, basically with bioactivity of other Berberis taxa must be consulted. Moreover, the authors probably missed to see the Academic Editor's comments in the previous round, since they were left unaddressed as well. I will repeat them:

"The authors have presented the whole manuscript in a rather indistinct fashion, gaps in the content are visible and the whole text lacks flow. Moreover, there are numerous places in the M&M section as well as in Results containing inconsistent methodology and vaguely presented results and conclusions. The manuscript would hugely benefit if being proofread by a senior researcher of the similar expertise, which would steer the authors how to properly present their experiments. It is also highly recommended to have the manuscript copyedited by a native English speaker or a professional editing agency towards gaining clarity and better readability."

Therefore, language polishing is much needed throughout the text. In L27 please do not introduce the abbreviated plant species name in parenthesis. It is a common scientific practice to abbreviate the genus name once being introduced in full.

Response: As behalf of all co-authors, I would like to appreciate this referee due to thoughtful comments proposed by the peer review. After we revised the manuscript, those significant issues could be changed.

As compared with other Berberis taxa have already added in Discussion. Moreover, the methods and results section were adjusted to address your comment. Furthermore, the English revision in the revised manuscript have already performed by Editage Company. Finally, the abbreviated plant species name in parenthesis have already removed in the revised manuscript.

**Response to reviewer #1**

General comments: nothing special, ,most of the corrections are addressed but language still needs serious revision. In my first review, I strongly suggested to edit language by a native English speaker.

Response: Thanks for this suggestion, and the English revision is provided by the Editage Company.

**Response to reviewer #3**

General comments: The authors have incorporated most of the suggestions made by the reviewers. However the following items must be improved:

Response: As behalf of all co-authors, I would like to appreciate this referee due to thoughtful comments proposed by the peer review. After we revised the manuscript, those significant issues could be changed.

Question 1: Lines 344-346: “…Our study found the anthocyanin content in B. heteropoda fruit was inconsistent with a prior study [3], which indicated the TAC was 20.37 mg/g fresh weight of B. heteropoda...”. Are the differences between 19.83 and 20.37 mg cyanidin-3-glucoside equivalent/g fresh weight significant?

Response: Thanks for this suggestion. According to the standard deviation of TAC in our study and prior study, we noted the difference was associated with statistically significant.

Question 2: As a general comment of the Discussion section, the authors do not discuss the obtained results with other Berberis fruits species, in particular with respect to TPC, TFC and TAC. New bibliography on other Berberis species were not included in the revised manuscript.

Response: Thanks for this suggestion, and several sentences have already added in Discussion section to compare the obtained results with other Berberis fruits.

Question 3: Final comment: this manuscript needs to incorporate the corrections suggested before its publication in PLOS ONE.

Response: Thanks for this suggestion, and all of changes have already performed to address your comments.
